# Supplementary material for: Mitigation of Breast Cancer Cells’ Invasiveness via Down Regulation of ETV7, Hippo, and PI3K/mTOR Pathways by Vitamin D3 Gold-Nanoparticles
Source: Int J Mol Sci. 2024 May 14;25(10):5348. doi: 10.3390/ijms25105348 (PMC11120902; doi:10.3390/ijms25105348)
Supplement: Supplementary file 1 [file ijms-25-05348-s001.zip › ijms-2869655-supplementary.pdf]

Supplementary Materials

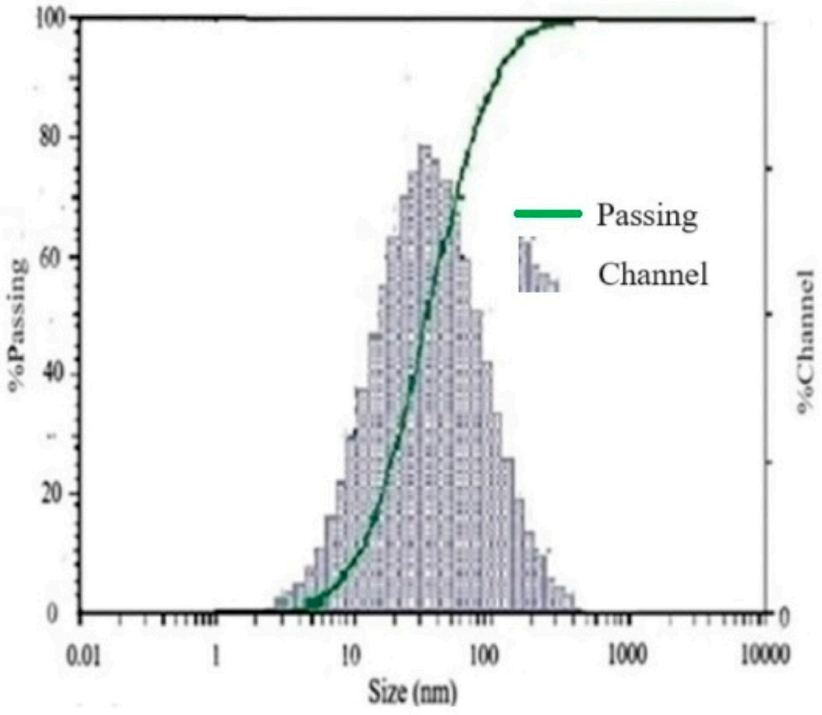

Supplementary Figure S1: The VD3-GNP DLS measurement size distribution chart

Expression pattern of key proteins in breast invasive cancer patients:

During BC the levels of (Normal n=114 vs primary tumor n=1097) AKT (p<1E-12), MTOR (p<5.98E-02), and ETV7(p<1E-12) proteins are upregulated significantly (Supplementary Figures S2B-D), while PI3k is down regulated (p<1.63E-12) (Supplementary Figure S2A). We used data from the 'The Cancer Genome Atlas Program (TCGA) database and UALCAN software [52,62]. In the Hippo pathway YAP is downregulated (p<1E-12) (Supplementary Figure S2E) and TAZ is upregulated (p<1.25E-11) (Supplementary Figure S2F).

Both YAP and PI3K proteins are downregulated in cancer, though the active phosphoproteins (not quantified) lead to cancer metastasis.

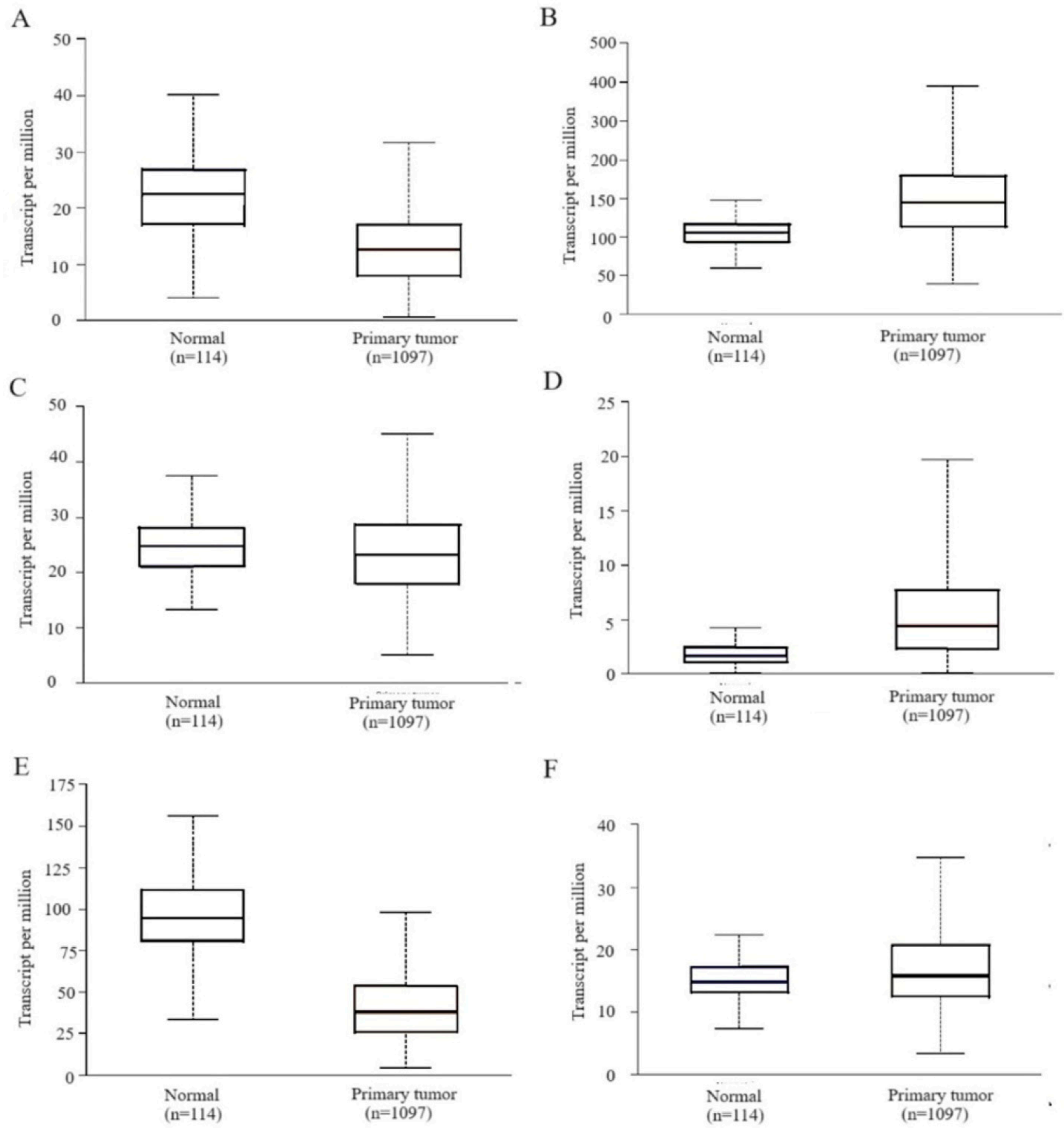

Supplementary Figure S2: (A-F) Expression profile of (PI3K, AKT, MTOR, ETV7, YAP, TAZ) in patients from TCGA database (using UALCAN software) (Normal vs primary tumor n=114, n=1097)

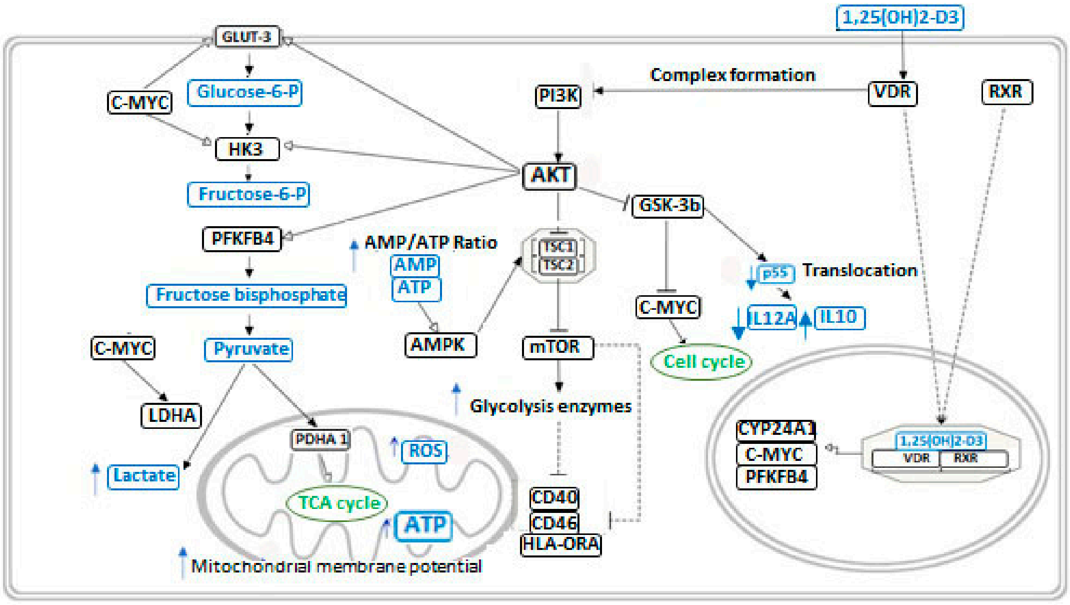

Supplementary Figure S3: KEGG pathway of Vitamin D action.

The KEGG pathway (Supplementary Figure S3) shows that Vitamin D binds with the VDR and acts on PI3K/AKT/MTOR. Interestingly, we find that VD3-GNP treatment also downregulates ETV7 expression and YAP, TAZ in BC cells which are not in the KEGG pathway. Based on our own findings we propose a mechanism of VD3-GNP reduction of cancer cells' aggression as depicted in the graphical abstract.

Network details

In our current protein network modeling the Network nodes representing proteins, splice isoforms, or post-translational modification are collapsed, i.e., each node represents all the proteins produced by a single protein-coding gene locus.

Node color colored 'ball shaped nodes' are proteins in the first shell of interactors; white nodes are in the second shell of interactors. Node content: Empty nodes are proteins of unknown 3D structure; filled nodes indicate that the 3D structure is known or predicted. Edges represent protein-protein associations. Edge colors: curated database (cyan edge); experimentally determined (pink edge); predicted interactions: gene neighborhood (green edge); gene fusions (red edge); gene co-occurrence (blue edge). Others: text mining (yellow edge); co-expression (black edge); protein homology (blue edge).

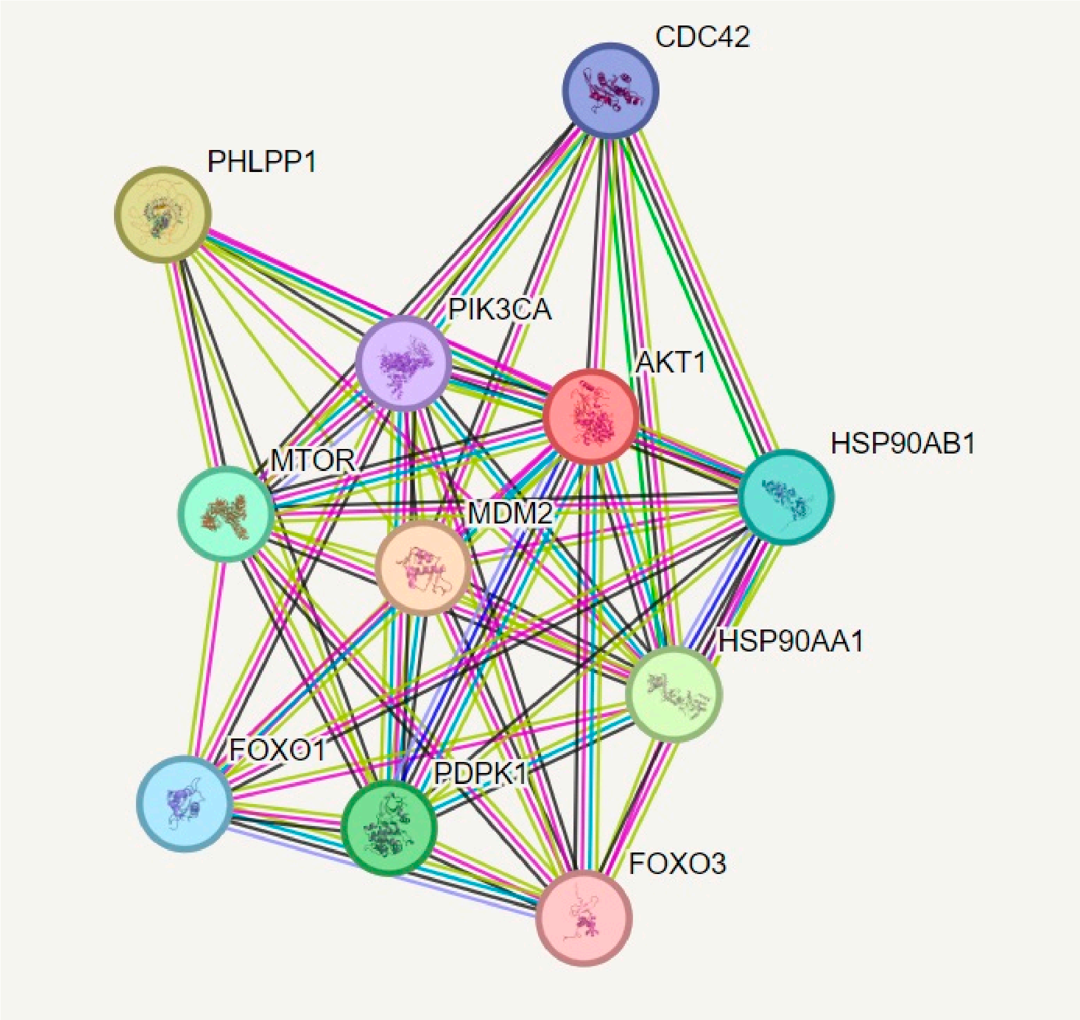

Supplementary Figure S4: (A) PPI diagram for AKT1.

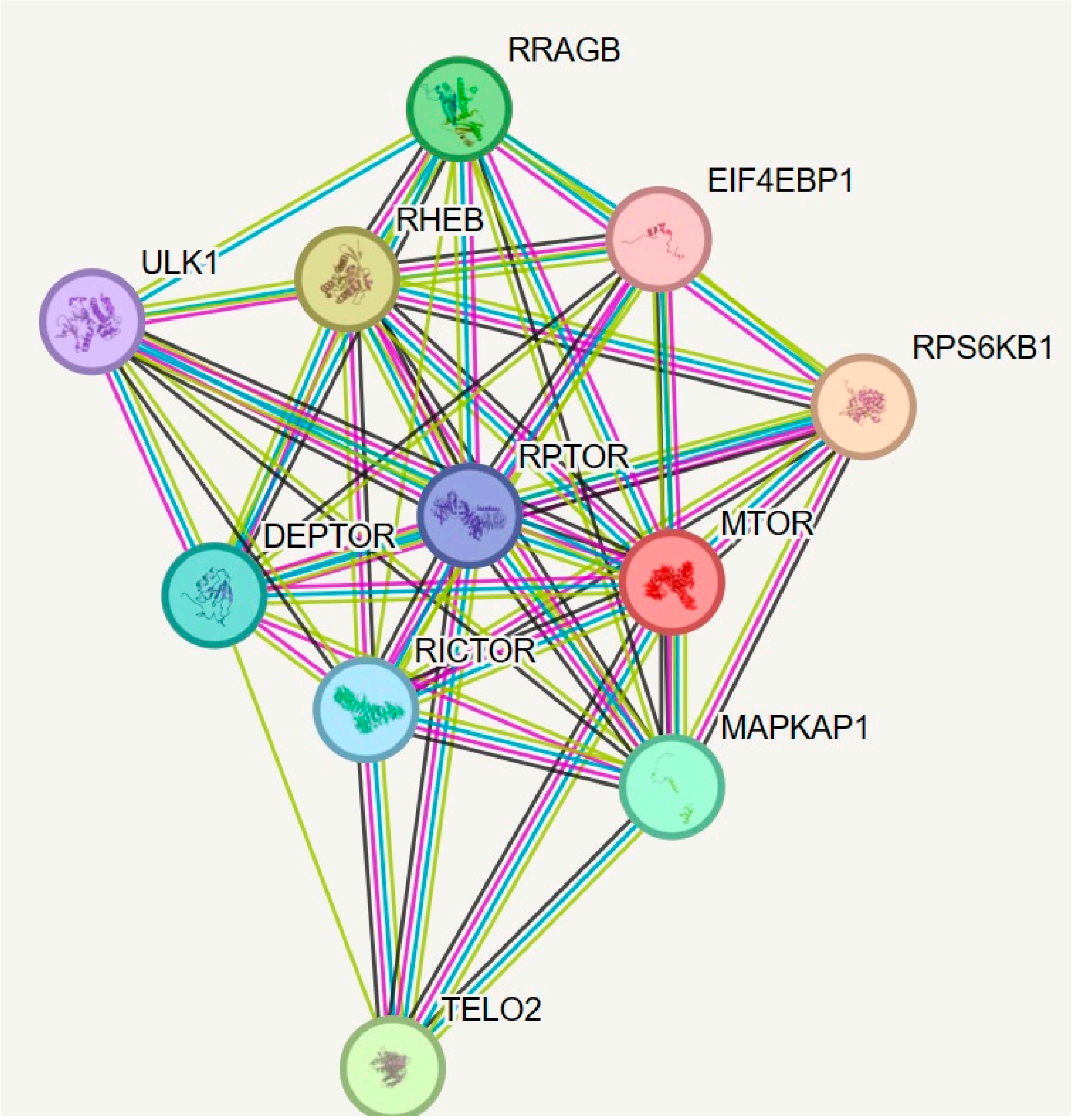

Supplementary Figure S4: (B) PPI diagram for MTOR.

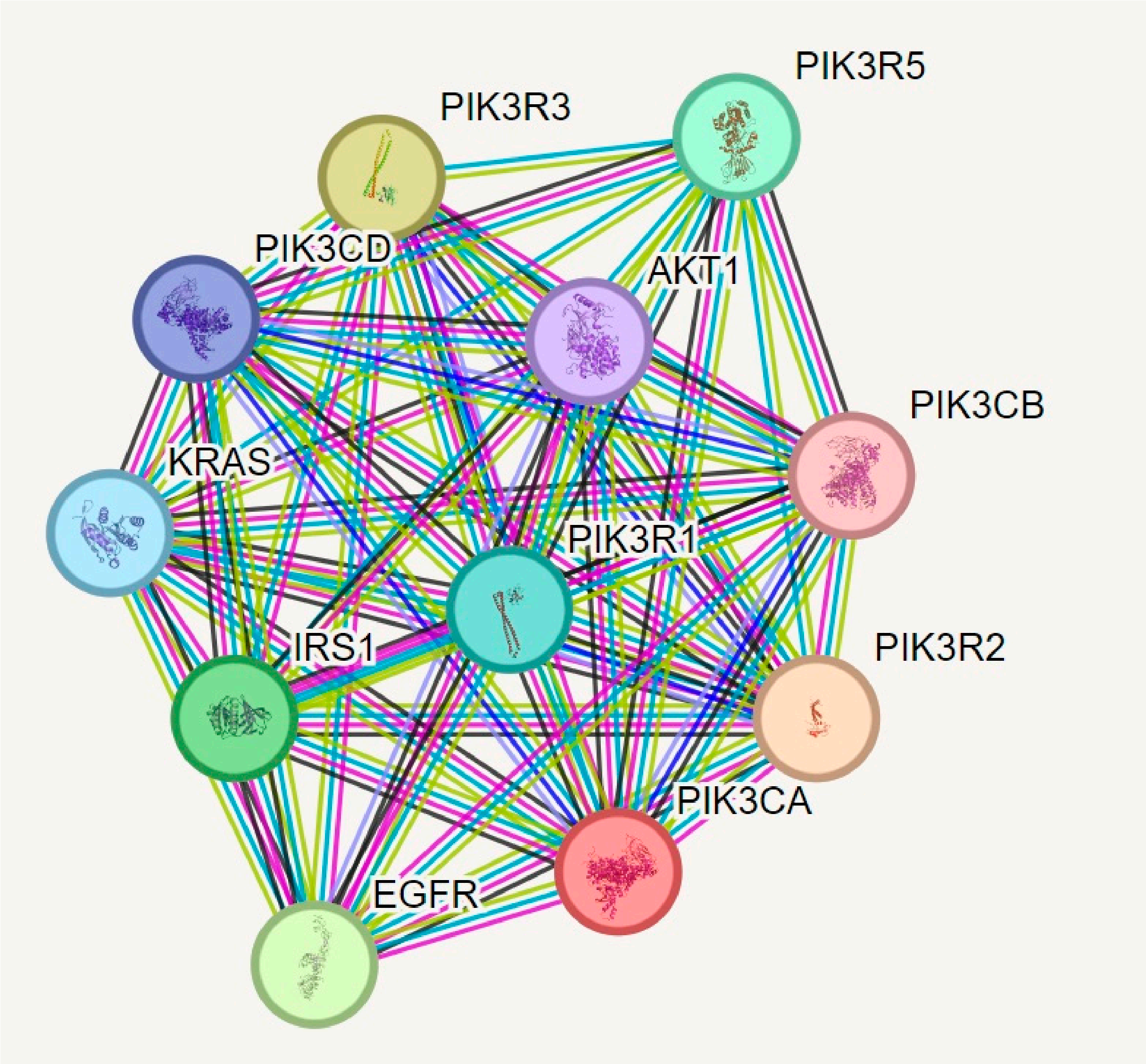

Supplementary Figure S4: (C) PPI diagram for PI3K.

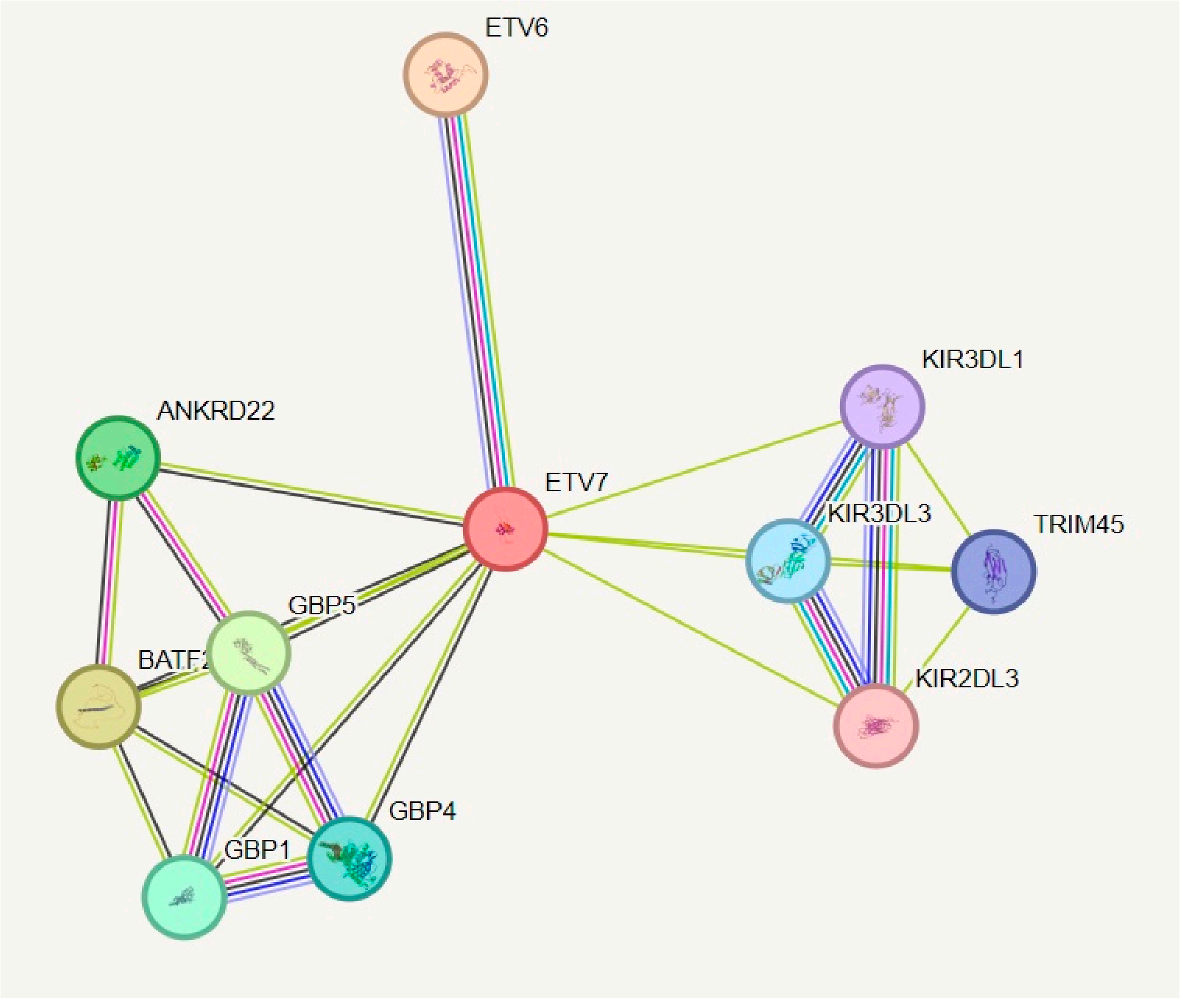

Supplementary Figure S4: (D) PPI diagram for ETV7.

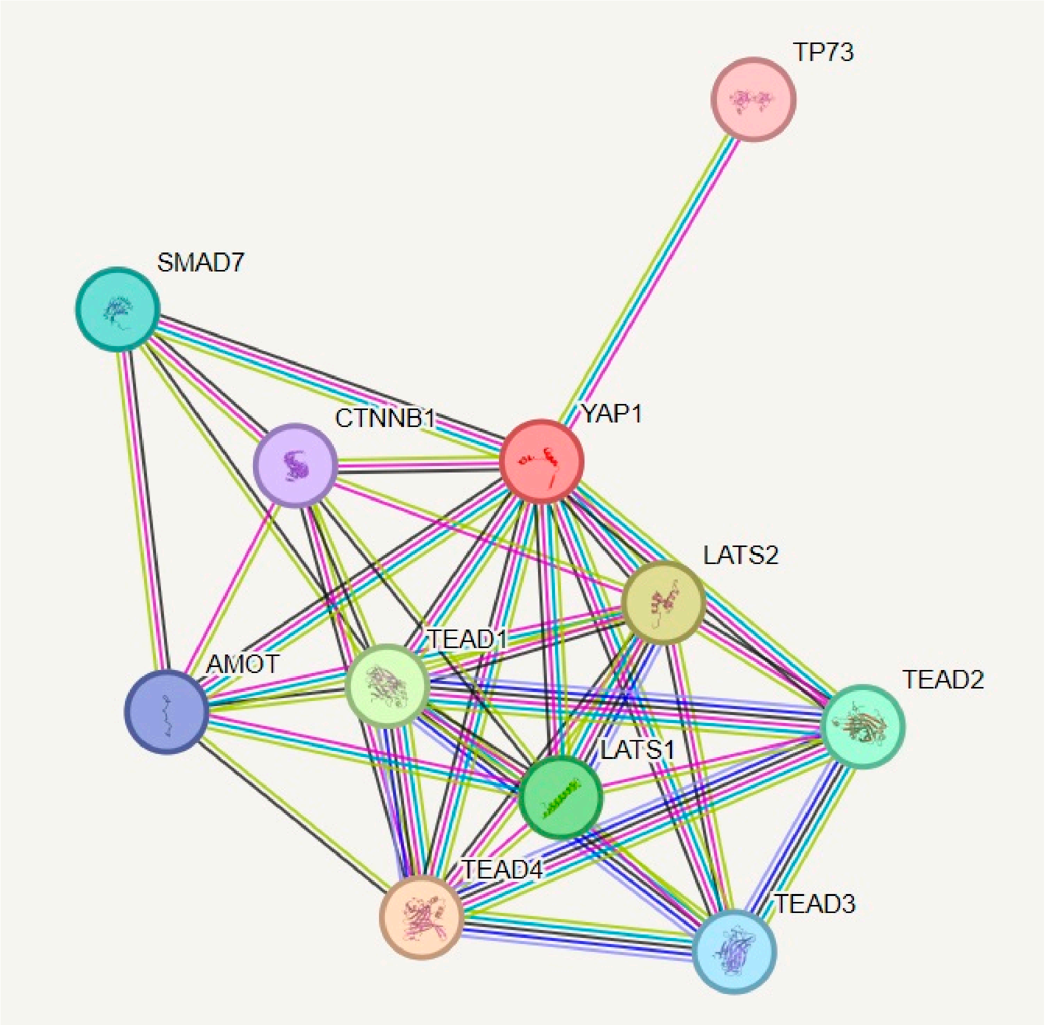

Supplementary Figure S4: (E) PPI diagram for YAP.

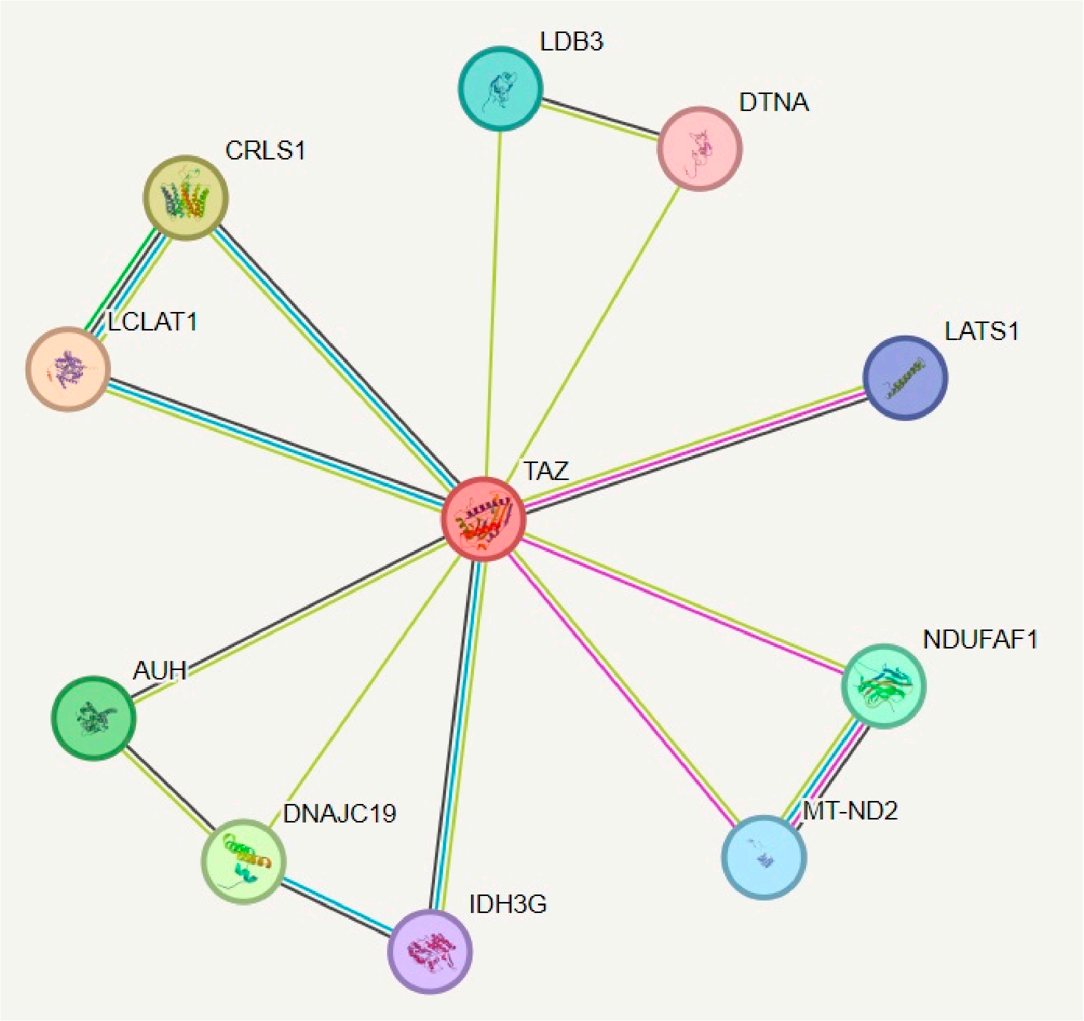

Supplementary Figure S4: (F) PPI diagram for TAZ.
